# Tuna labels matter in Europe: Mislabelling rates in different tuna products

**DOI:** 10.1371/journal.pone.0196641

**Published:** 2018-05-16

**Authors:** Carmen G. Sotelo, Amaya Velasco, Ricardo I. Perez-Martin, Kristina Kappel, Ute Schröder, Véronique Verrez-Bagnis, Marc Jérôme, Rogério Mendes, Helena Silva, Stefano Mariani, Andrew Griffiths

**Affiliations:** 1 Instituto de Investigaciones Marinas (CSIC), Vigo, Spain; 2 Max Rubner-Institut (MRI), Department of Safety and Quality of Milk and Fish Products, Hamburg, Germany; 3 Ifremer, Nantes, France; 4 Portuguese Institute for the Sea and Atmosphere (IPMA, I.P.), Lisbon, Portugal; 5 Ecosystems & Environment Research Centre, School of Environment & Life Sciences, University of Salford, Greater Manchester, United Kingdom; 6 Biosciences, University of Exeter, Exeter, United Kingdom; Universita degli Studi di Bari Aldo Moro, ITALY

## Abstract

Tuna fisheries and processing represent economic activities of paramount importance around the world. Most of these products are traded for human consumption and in general are highly demanded commodities. However, not all tuna products achieve the same market price, some consumers are willing to pay a huge amount of money for certain species (i.e. Japanese market for Bluefin tuna) while other species are rather affordable (i.e. Skipjack tuna), therefore mislabelling has been observed frequently. We collected and analysed 545 tuna samples in six European countries, including fresh, frozen and canned products, and we have investigated whether or not these products were correctly labelled under European and national legislations. We found an overall mislabelling rate of 6.79%; in particular, 6.70% of the fresh and frozen tuna products and 7.84% of canned tuna were mislabelled, and only in the case of fresh and frozen tuna samples significant differences among countries were found. Mislabelling rates for Atlantic Bluefin tuna labelled products were very high, ranging from 50 up to 100%. In general, mislabelling was higher when specific names were included in the labels. The “tuna” umbrella term is a very popular one with consumers, but also one that remains vulnerable to ambiguity, hampering efforts towards market transparency and with potential negative consequences to the adequate management of tuna species stocks.

## Introduction

Seafood fraud is more common than most consumers think and many studies have highlighted the fact that species substitution is especially frequent in certain seafood products, such as those labelled as Atlantic Bluefin tuna, European hake or Atlantic cod [1–3]. The consequences of this malpractice not only involve the economic deception of consumers [4], but may also have a negative impact on the sustainability of marine resources [5]. However, the diversity and number of fish commonly traded globally as seafood is so vast, that much remains to be done to understand the true ecological costs of mislabelling [6].

One of the essential elements in the fight against seafood fraud is legislation. European Union (EU) labelling regulations are aimed at providing information to consumers such as commercial and scientific names, thus assuring their traceability and identification throughout the value chain (EU 1379/2013), however in this regulation the type of fishery or aquaculture product determine the mandatory information required in the labels and, therefore, may decrease the expected effects [7]. Recent studies suggest that seafood mislabelling has generally decreased in European countries due to the existence and enforcement of these labelling regulations and the use of appropriate species identification methodologies [1,8]. This can also be linked to the EU involvement in funding projects dealing with this problem from the very beginning of the EU framework programme [9], putting Europe at the forefront of the authenticity tests development, especially regarding seafood [10].

Tunas are among the most desirable marine fish worldwide, with a global tuna and tuna-like species catch that peaked at 7.7 million tonnes in 2014 [11]. Skipjack (*Katsuwonus pelamis*) and Yellowfin (*Thunnus albacares*) were the tuna species most captured with about 3 and 1.5 million tonnes, respectively [10]. In contrast, the captures of Bluefin tuna (three species: *T*. *thynnus*, *T*. *orientalis* and *T*. *maccoyii*) during the same period did not exceed 40,000 tonnes. The conservation status of the different tuna species and stocks is also variable but worrying: several stocks are overfished (31%) and near to that threshold (17%), whereas 52% remain at a healthy level of abundance [12]. However, market demand has not decreased and the tuna fishing fleet maintains their capacity [10].

Atlantic Bluefin tuna (*T*. *thynnus*) deserves special attention since the strong market demand on this species during the last decades nearly provoked a collapse in the populations, such as East Atlantic and Mediterranean, which forced the reduction of the total allowable catch (TAC) for the Mediterranean fishery since 2007. This measure allowed the recovery of the stock [13]. Since the most desirable species are not always available for the market, strong economic forces may result in some degree of substitution, fraud or mislabelling [14]. It is not easy to find a global mislabelling rate for tuna; different studies have shown different levels. In general, factors such as country, type of retailer, sampling target or year may explain these differences. Pardo et al. [15] suggest an average 18% misdescription for tuna, Gordoa et al. [16] found 37% of fresh and frozen tuna in Spain at points of sale and 48% in restaurants. In some other cases, these values were extremely high such as 95% found by Oceana in Brussels restaurants for Bluefin tuna [17]; however, studies have typically varied in their sampling strategies, and therefore remain poorly comparable.

Transnational evaluation of seafood fraud could reveal trends among countries or geographic areas, which ultimately could help to design coordinated measures to reduce the global incidence of mislabelling. However, transnational studies are scarce; some examples were reported in North America, with samples taken from USA and Canada [18]. As part of the Labelfish project, 1563 seafood samples of different categories and processing degrees were collected across 19 cities and six European countries, revealing an average mislabelling rate of 4.93% for the European retail sector [7]. Later, in 2015 the EU Commission organised a coordinated plan to analyse 3906 samples of fish, mostly white fish, in 27 Member States and 2 EFTA (European Free Trade Association) Member States. These samplings and analyses resulted in identifying an overall mislabelling of white fish in Europe of 6% [19].

This study benefits from the previous Mariani et al. [7] sampling, focusing only in the tuna products. The objective was to gain deeper understanding of the patterns and drivers of tuna mislabelling across Europe by examining the factors affecting mislabelling rates of these products in six European countries. In particular, the analysed factors included the influence of processing and species labelling in mislabelling rates, and the type of substitution which characterizes the fraud in tuna products.

## Materials and methods

### Sampling

Commercial samples of tuna products were purchased in markets of 18 different cities in Europe belonging to 6 countries (France, Germany, Ireland, Portugal, Spain, and United Kingdom) between 2013 and 2014. Locations were chosen in order to have a good coverage and geographical replication for each country (S1 Table).

In each city, the sampling was aimed to cover a wide metropolitan area and a wide range of types of retailers, including supermarkets, traditional markets and specialized fishmongers. When several products were purchased in a single store these were chosen with different brands or types of processing. The most abundant types of tuna products (fresh, frozen and canned tuna) have been sampled in all countries, while in some Southern regions in Europe (in Spain, Portugal and France), other types of convenience food containing tuna were also sampled and analysed, such as salads and precooked products. 545 samples were successfully analysed: 225 were fresh and frozen (unprocessed), 268 canned (processed) and 52 miscellaneous (processed). The number of samples analysed per country were: 87 in Spain, 71 in Portugal, 93 in France, 53 in ROI, 154 in UK, and 87 in Germany (S1 Table).

Samples were obtained in their original packaging and were transported to the laboratory on the day of purchase, where they were stored at -20°C, or a small piece of tissue was removed and preserved in absolute ethanol. Packaging was retained or photographed and all label information was registered.

### Assessment of compliance with European seafood labelling legislation

Determination of tuna commercial products mislabelling was carried out taking into consideration the adequate European regulation:

- (EC) No 852/2004 where it is established the definition of processed and unprocessed food (i.e. fresh and frozen tuna fall into the category of unprocessed fishery and aquaculture products, while canned tuna into the processed ones).
- EEC 1536/92 where it is stated that preserved tuna and bonito must be prepared exclusively from certain fish species (i.e. tuna cans should contain only any *Thunnus* species or *Katsuwonus pelamis*), mixing of species is not allowed in each tin (unless muscular structure has disappeared), only commercial names are required
- EU1379/2013 indicating the required information to be presented to consumers, among others commercial and scientific names (the latter except for canned and other prepared products such as salads).
- Also, all member states have translated these European regulations into national legislation, the main aspect to be considered is the specific denomination that each country establishes to designate different tuna products, including fresh, frozen and canned tuna. A summary of these denominations is presented in S2 Table.

### DNA extraction, amplification and analysis

Sample screening involved five European laboratories with extensive experience in seafood authenticity. Each lab also carried out blind-sample ring trials to ascertain consistency of the methods used to identify fish species (full details of these experiments are presented in S1 File).

A summary of the methods employed in this study is presented in S3 Table, where the specific DNA extraction commercial kits, primers used, molecular regions and size of the amplicons, DNA sequencing procedure and sequence analysis are indicated.

Different mitochondrial DNA target regions were used in the laboratories involved in the study: cytochrome b, cytochrome oxidase I and control region. Tuna species in fresh and frozen products were identified using larger fragments, 464 bp for cytochrome b marker, 650 bp for cytochrome oxidase I and 450 bp for the control region marker [20,21]. In the case of canned tuna most laboratories used cytochrome b markers of shorter size, 187 and 176 bp [9,22], except for France, which used a short control region fragment of 150 bp [8].

Primers and PCR protocols are specified in S4 Table. Once checked by agarose electrophoresis in 1–2% agarose gels, PCR products were sequenced. Subsequent DNA sequence analysis was performed as indicated in S3 Table. Briefly, sequences were edited using Chromas (Technelysium), Bioedit [23] and GeneDoc [24] and matched against NCBI database using the nucleotide BLAST (Basic Alignment Search Tool) and the BOLD (Barcode of Life) database for the COI sequences [25]. Species were identified using a 99% minimum match criterion, with the exception of *Thunnus albacares* and *T*. *obesus*, where the threshold was 100% [26]. For an unequivocal identification, laboratories from Spain, France and Germany also constructed Neighbor-Joining trees using MEGA software [27] with their own reference sequences using Tamura-Nei distances.

Sequences longer than 200 bp were uploaded in GenBank (accession numbers: Germany KJ531289 to KJ531379; France KJ535741 to KJ535783; Spain KJ623816 to KJ623830; Portugal MF067430 to MF067499) and BOLD database in the case of COI sequences (UK and ROI KJ510424 KJ531384 and KJ563141-KJ645864)

Samples containing a different species than the one declared in the label were considered mislabelled. When only the commercial name was present, mislabelling records were obtained by following each country’s list of approved commercial designation for tunas (S2 Table). When the scientific name was present, it was the one taken as a reference to compare with the analytical result.

### Statistical analysis

Software GraphPad Prism was used to perform pairwise comparisons between data sets using Chi-square with Yate’s correction and correlation analysis for the dependence between number of samples and mislabelling rate.

### Results and discussion

**Fig 1:**
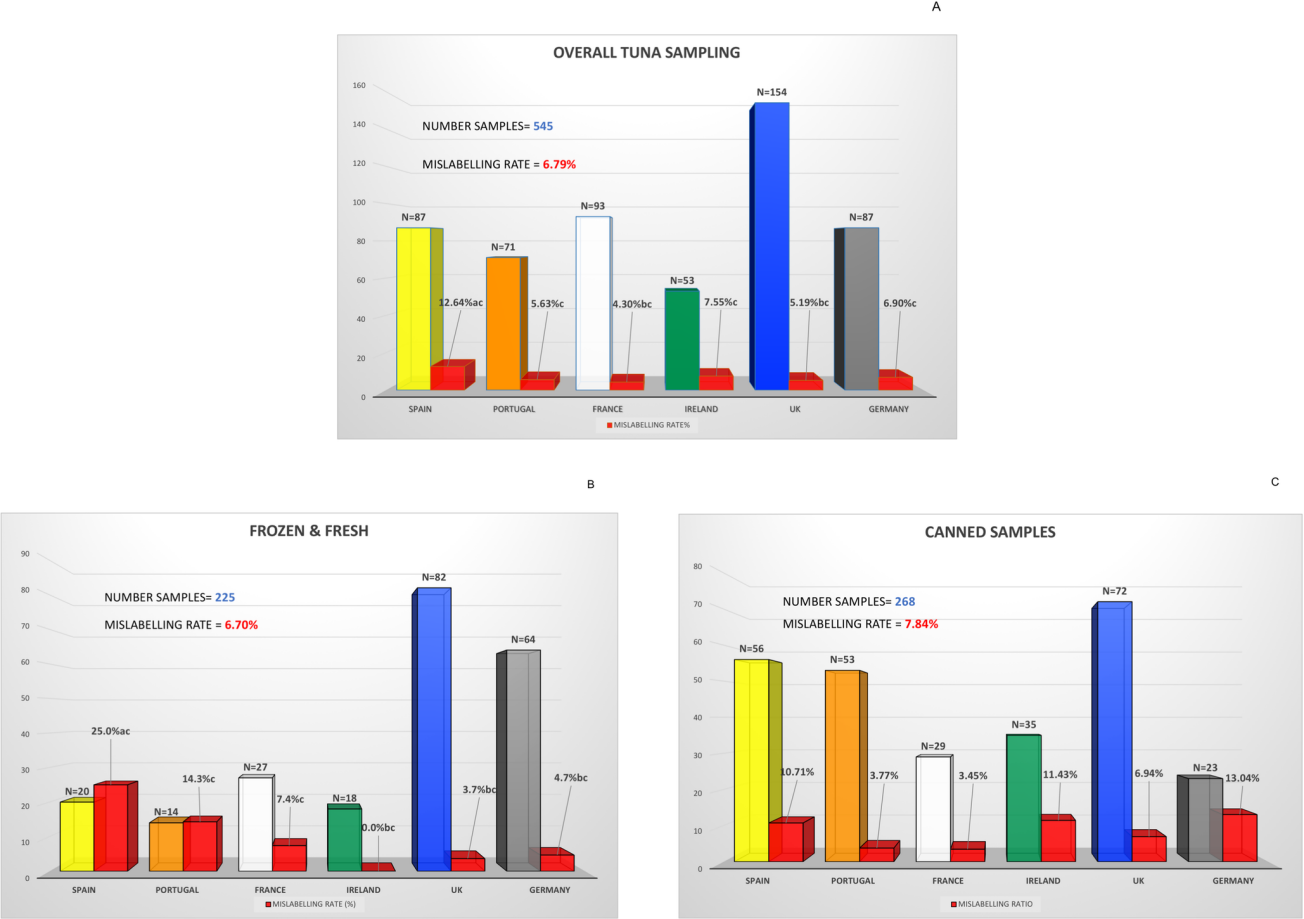
Collected samples and mislabelling rate for tuna seafood products across six European countries. Fig 1A: Number of collected samples and mislabelling rates (red bars) for all analysed tuna products in six European countries. Statistical differences are shown by letters, different letters indicate P<0.05. Fig 1B: Number of collected samples and mislabelling rates (red bars) for fresh and frozen tuna products in six European countries. Statistical differences are shown by letters, different letters indicate P<0.05. Fig 1C: Number of collected samples and mislabelling rates (red bars) for canned tuna products in six European countries.

545 tuna samples–of which 225 fresh & frozen, 268 canned and 52 miscellaneous (e.g. dried, roes, salads etc.)–were successfully sequenced and identified (for more detailed information about the results of previous tuna species identification ring trials see S1 File); 37 of them were mislabelled, making an overall mislabelling rate of 6.79% (Fig 1). This study is not only the largest sampling effort reported for tuna products but also one that involves six different European countries. Previously reported mislabelling rates for tuna were very variable, on average 18% [15], mainly because these rates may change over time and also because the type of retailer or provenance of samples influence dramatically the results obtained. The much lower value found here may be an indication of the impact of labelling legislation and control, the mass media coverage of food fraud with the consequence of consumer being more aware of food labels [8].

The mislabelling rate was not significantly different (Chi-square = 0.1045; df = 1; P = 0.3733) between fresh & frozen and canned tuna, 6.70% and 7.84%, respectively, while the miscellaneous products showed 1.92% mislabelling (not significative differences among them). Previous works have reported higher mislabelling rates, recently Gordoa et al. [16] have shown that fresh and frozen tuna in Spain was mislabelled up to 37% at points of sale, while in restaurants the mislabelling rate was even higher, 48%. In a previous report from Oceana [28], tuna samples obtained at different points of the value chain in different states of the USA showed that 58% of tuna products were mislabelled. However, this overall data may not be comparable directly, because the specific type of tuna products (i.e. species) have a relevant influence on the final mislabelling rate.

Fig 1A shows that small differences between mislabelling rates were observed among Portugal, France, Ireland, UK and Germany (not significant), with France showing the lowest mislabelling rate of the study. On the other hand, Spain showed the highest mislabelling rates, with significant differences with those of France and UK (Chi-square = 3.0760; df = 1; P = 0.0397 and Chi-square = 3.2840; df = 1; P = 0.0350, respectively). However, these overall mislabelling rates did not reflect the mislabelling situation of different tuna products: i.e. while some products exhibited 1.92% mislabelling rate (for example the category “miscellaneous”), fresh and frozen tuna products were mislabelled at 6.70% (Fig 1B). In fact, we have observed significant differences for fresh and frozen tuna for Spain (25%) compared with Ireland (0%, Chi-square = 3.2250; df = 1; P = 0.0363), UK (3.7%, Chi-square = 7.3940; df = 1; P = 0.0033) and Germany (4.7%, Chi-square = 5.1300; df = 1; P = 0.0118). Canned samples presented an overall mislabelling rate of 7.84%, in this case differences in canned tuna among countries, ranging from 3.45% (France) to 13.04% (Germany) were not significant (Fig 1C).

Additionally, no significant correlation was found between the number of samples taken in each country for each category or the seafood consumption ratio per habitant and the mislabelling rate (data not shown), therefore other factors should be considered when interpreting the results obtained.

**Fig 2:**
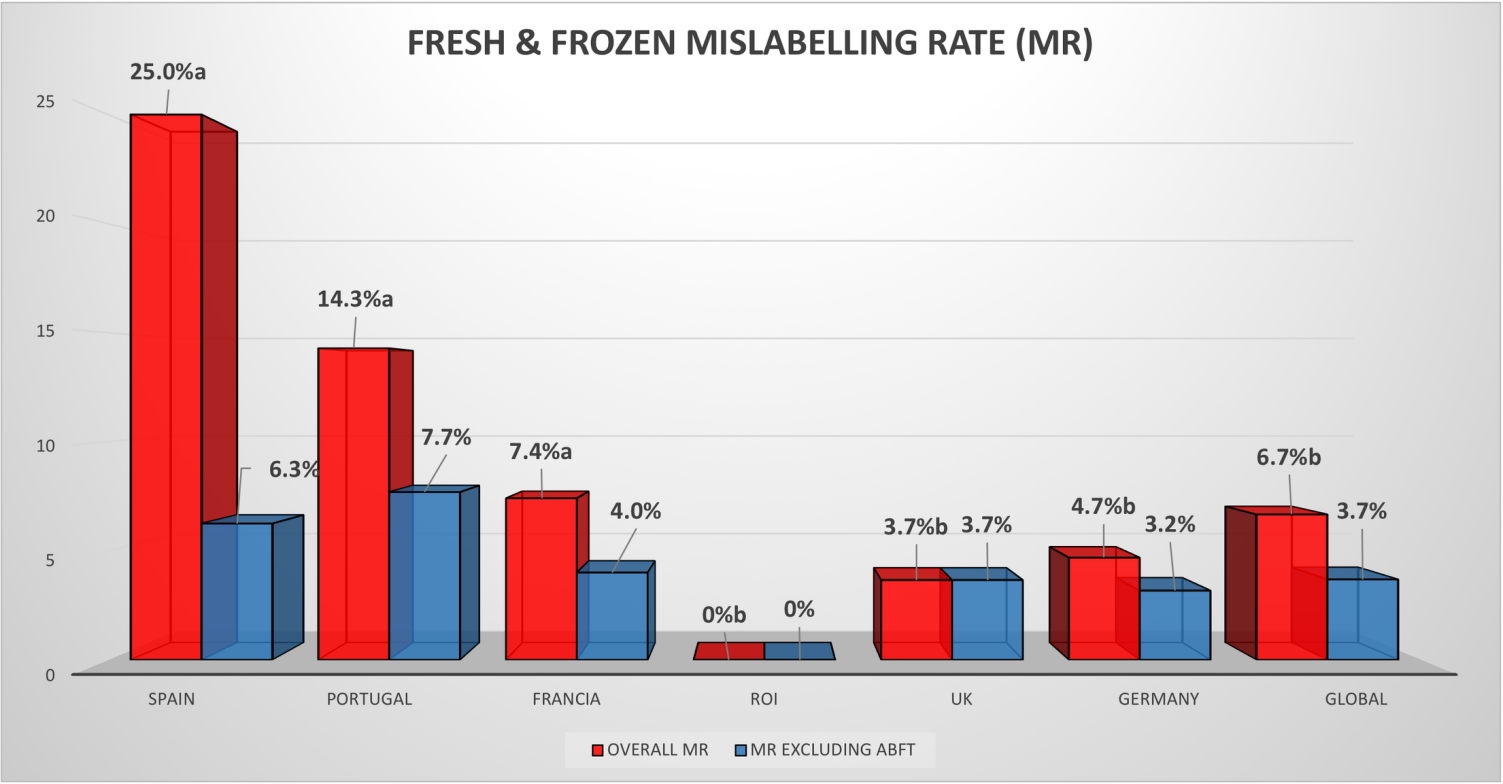
Mislabelling rate for fresh and frozen tuna seafood products across six European countries. Overall mislabelling rates (OVERALL MR) for fresh and frozen tuna products and mislabelling rate of products excluding those labelled as Atlantic Bluefin tuna (MR EXCLUDING ABFT) in six European countries. Statistical differences are shown by letters, different letter indicate P<0.05.

The analysis of the influence of the type of labelling in the final mislabelling rate can be observed in Fig 2 and S5 Table. Although a significant correlation couldn’t be established among the number of samples labelled as tuna and the mislabelling rate in a particular country (data not shown), the influence of sampling tuna product with Atlantic Bluefin tuna label can be clearly seen in the changes in mislabelling rates: when these products are excluded in the mislabelling rate calculation, the differences among countries were not significant. In fact, while overall mislabelling rate drops down to 1% in the case of labelling just tuna, it goes up (88%) in the case of Atlantic Bluefin tuna labelled products. These results agree with the results found by Vandamme et al. [29] that reported a low mislabelling rate for tuna in sushi of about 10%, but which rises up to 18% when tuna species is considered in the labelling. Likewise, Gordoa et al. [16] reported a 73% of mislabelling for Atlantic Bluefin tuna and points towards economic gain as the main reason for mislabelling tuna. Atlantic Bluefin mislabelling has been highlighted as an example of inverse relationship between low volume catches and high proportion of substitution; offer and demand do not match and the result is a very high mislabelling rate [30].

**Fig 3:**
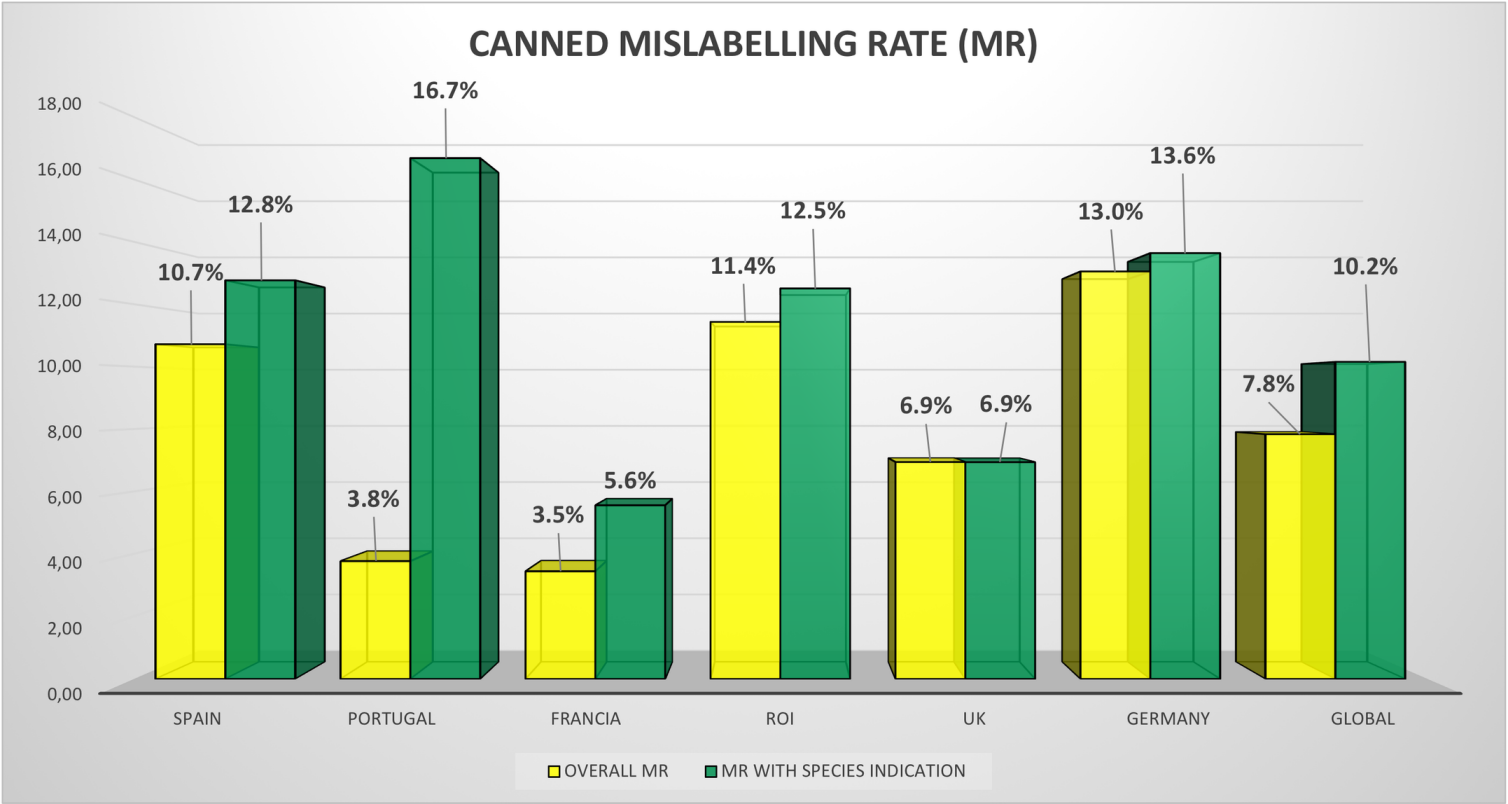
Mislabelling rate for canned tuna seafood products across six European countries. Overall mislabelling rates (OVERALL MR) for canned tuna products and mislabelling rate of products with labels indicating species (MR INCLUDING SPECIES) in six European countries.

In the case of canned tuna, including species in the label provoked an increase in the mislabelling (Fig 3 and S6 Table), and significant differences could be observed in overall values, from 1% mislabelling rate for Tuna labelling up to 10% mislabelling rates when species are indicated in the label (Chi-square = 4.381, p = 0.0182). General names such as tuna, which include any species of the genus *Thunnus* and *K*. *pelamis*, were associated with very low mislabelling rates (0%), while indicating the species in the can resulted in a higher mislabelling rate (17–60%) (S6 Table). In general, these mislabelling rates were lower than those found for fresh or frozen tuna products.

Current labelling legislation in EU establishes the obligation to indicate commercial name and species in the case of fresh, frozen, smoked and dried seafood products (EU1379/2013). However, we have found many fresh and frozen tuna products, from 29% in Portugal up to 100% in Ireland, which were still labelled with the generic name tuna (S5 Table). Although we did not consider them mislabelled in this study, these results indicate a poor implementation of labelling rules across Europe. In the case of canned products, legislation allows the use of generic names, such as tuna, and as it can be seen here, samples with the generic name tuna have shown very low mislabelling rates. These differences in labelling requirements between fresh/frozen and canned tuna are difficult to understand from a consumer point of view as the same need for information should be required. In fact, the campaign “one fish, one name” advocates for the establishment of more specific names for every type of seafood product [31] and the main objective is to empower consumers to make more accurate purchasing decisions. The implementation of this approach will help to better protect resources and to fight illegal fishing practices.

**Fig 4:**
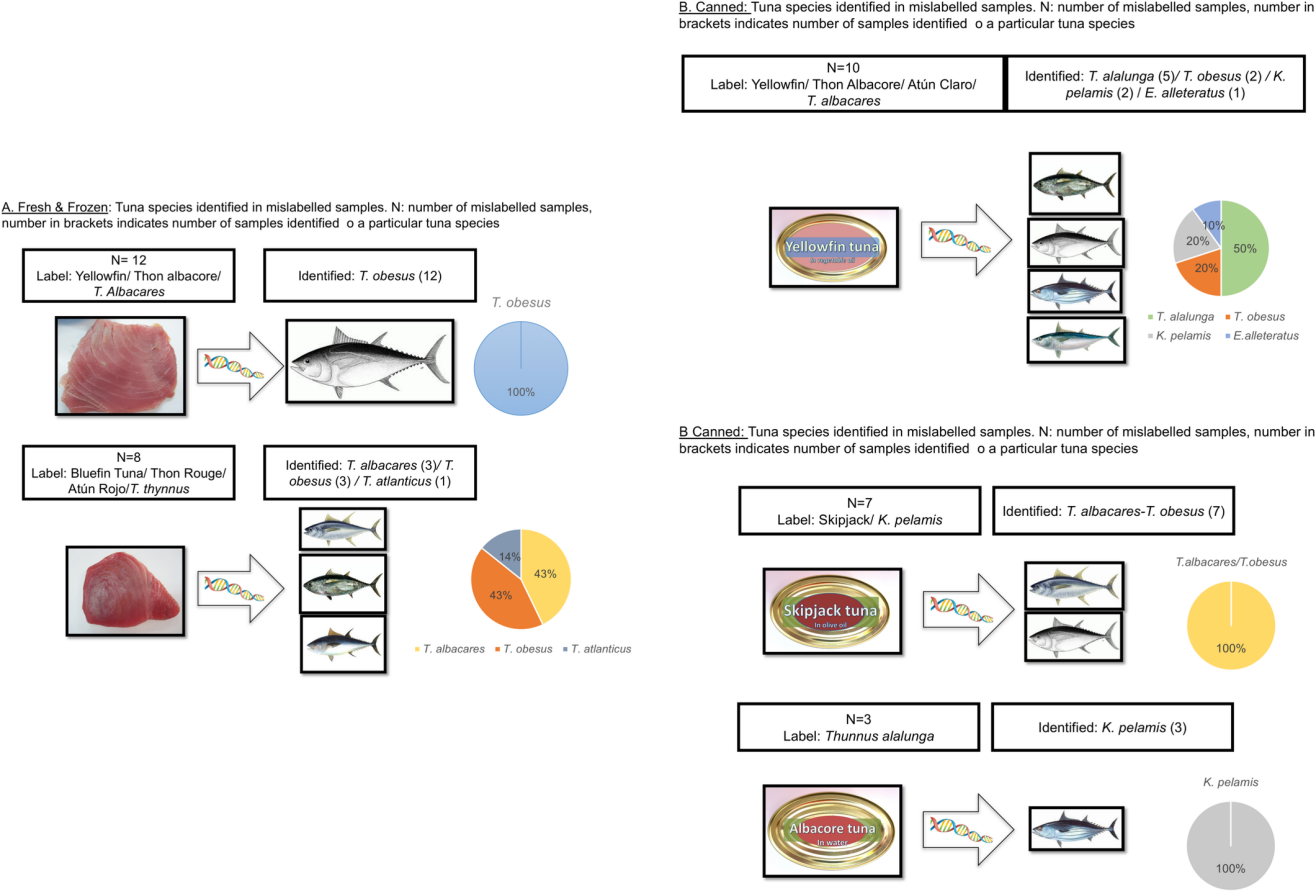
Tuna species identified in mislabelled samples. Fresh and frozen: tuna species identified in mislabelled fresh and frozen tuna samples. Canned: tuna species identified in mislabelled canned tuna samples. N: number of mislabelled samples, number in brackets indicates the number of samples where a particular tuna species was identified.

Fig 4 and S7 Table show the type of substitution observed in the analysed samples. Frozen and fresh samples exhibited two main types of substitutions, affecting the products labelled as *T*. *albacares* and *T*. *thynnus*. In the case of *T*. *thynnus* (7 samples mislabelled), commercially labelled as Atlantic Bluefin tuna, thon Rouge or atún Rojo, the levels of substitution by *T*. *albacares* or *T*. *obesus* were similar (43%). More mislabelled samples were found for *T*. *albacares* (Yellowfin, thon Albacore, atun de aleta amarilla), in this case only one species, *T*. *obesus*, was found to substitute this species. In this latter case, unintentional substitution may take place due to occurrence of mixed schools of both species, which are very similar as juveniles [32], and can be captured at the same time.

In terms of species, substitution results in canned tuna were different from those of fresh and frozen tuna. Canned tuna labelled as Yellowfin, Light tuna, thon albacore and atún Claro were replaced by *T*. *alalunga* (50% of samples, all of them from Spain), *T*. *obesus* (20%, none of them were samples from Spain, national legislation allows in this country to label *T*. *obesus* as atún Claro) and *K*. *pelamis* (20% of the samples) and *E*. *alleteratus* (10%). This is an example of lack of coherence in the European legislation: in one country, it is permitted to label *T*. *albacares* or *T*. *obesus* as Light tuna (Spain), while in the other European countries is not allowed. One might expect that this may have an impact on the mislabelling rates found for Light tuna in Spain, however, mislabelling rates for this product were similar to other countries where only *T*. *albacares* can be used in Light tuna can production. The results shown in S6 Table indicate that percent of samples labelled generically as tuna has an impact in the mislabelling rate of the country for canned tuna, since this type of products present mislabelling rates close to 0%.

Samples of *K*. *pelamis* were only relevant in UK, Germany, France and Ireland, while in countries like Spain and Portugal is very difficult to find canned tuna labelled as *K*. *pelamis*. In the case of *K*. *pelamis* labelled cans bought in the UK, all mislabelled samples were substituted by *T*. *albacares/T*. *obesus*. This type of substitution has been referred by some authors as reverse substitution, a cheaper species is substituted by an expensive one, and that may be an indication of hiding a product from a IUU fishery practice [16]. Another substitution was found in Germany where *T*. *alalunga* was substituted by *K*. *pelamis*. These results may indicate that different value chains, fresh/frozen tuna and canned tuna rely in different providers of raw material, besides the cultural aspects of each particular market (i.e. Spanish consumers value light appearance meat in canned tuna while this feature may be not as relevant in other European markets).

It has been reported that identification of the *Thunnus* species with mtDNA markers may present some problems due to low interspecific variability and introgression issues.

One of the problems is the low genetic distance found between *T*. *alalunga* and *T*. *orientalis*. There is also a low percentage of specimens of Atlantic Bluefin Tuna (ABFT) which exhibit almost the same sequence as Pacific Bluefin Tuna (PBFT) using some mtDNA markers and vice versa [26]. However, in our samples there were not any of these cases (i.e. a sample labelled as ABFT but being identified as PBFT) and although the current legislation would consider this example mislabelled (fresh and frozen tuna) this problem does not affect our mislabelling results. There is also introgression between ABFT and *T*. *alalunga*, 2–3% of ABFT specimens showing mitochondrial sequences of *T*. *alalunga*, but again we did not find any samples with this situation. Therefore, we conclude that in the present study these issues are not affecting our mislabelling results.

After looking into tuna product labelling across a significant portion of the largest global player in seafood trade (i.e. the European Union), it is apparent that this high-demand and widely marketed food category epitomises all the key challenges of global seafood sustainability and traceability. First, most of the products rely on catches from distant and/or tropical waters and a range of processing avenues, all of which poses the logistic challenges of a long and complex supply chain. Secondly, “tuna” is one of the general “umbrella” terms under which many species with diverse biological traits continue to be traded, especially in geographic areas exporting high volumes of seafood to Europe [33]. Collectively, the opportunities for species substitution, be it for deliberately maximising financial gain or through sheer logistic errors/mismanagement, remain high; so does the exposure of consumers to a lack of transparency that prevents environmentally conscious purchasing decision and/or may result in inadvertent consumption of unhealthy products (e.g. high heavy metal content, [34]). Progress in this area can be achieved through a robust international policy of accurate species-level labelling and coordinated governance efforts.

## Supporting information

S1 Table Tuna samples collected in six European countries between 2012 and 2014. (DOCX)

S2 Table Commercial denominations of tuna in EU and six member states (ES, PT, UK, ROI, FR, GER). (DOCX)

S3 Table DNA analysis methods used in this study by the participant laboratories. (DOCX)

S4 Table Primers sequences and PCR protocols used in the study. (DOCX)

S5 Table Number of samples of fresh and frozen tuna analysed and mislabelling results split by type of label. (DOCX)

S6 Table Number of samples of canned tuna analysed and mislabelling results split by type of label. (DOCX)

S7 Table Species which appear as substitute in the mislabelled samples. (DOCX)

S1 File Tuna species identification ring trials. (DOCX)
