# Supplementary material for: Tuna labels matter in Europe: Mislabelling rates in different tuna products
Source: PLoS One. 2018 May 16;13(5):e0196641. doi: 10.1371/journal.pone.0196641 (PMC5955508; doi:10.1371/journal.pone.0196641)
Supplement: S1 Table — (DOCX) [file pone.0196641.s001.docx]

|  |  | Type of processing | | |  | |  |
| --- | --- | --- | --- | --- | --- | --- | --- |
| Country | City | fresh/frozen | canned | others | Total analysed | Mislabelling % | |
| Spain | Vigo | 7 | 21 | 2 | 30 |  | |
|  | Santiago | 5 | 19 | 3 | 27 |  | |
|  | Madrid | 7 | 10 | 3 | 20 |  | |
|  | Bilbao | 1 | 6 | 3 | 10 |  | |
|  |  | 20/5 | 56/6 | 11/0 | 87/11 | 12.6% | |
| Portugal | Lisbon | 7 | 37 | 2 | 46 |  | |
|  | Porto | 4 | 9 | 1 | 14 |  | |
|  | Faro | 3 | 7 | 1 | 11 |  | |
|  |  | 14/2 | 53/2 | 4/0 | 71/4 | 5.63% | |
| France | Nantes | 5 | 15 | 19 | 39 |  | |
|  | Marseille | 22 | 14 | 18 | 54 |  | |
|  |  | 27/2 | 29/1 | 37/1 | 93/4 | 4.30% | |
| Republic of Ireland | Dublin | 9 | 17 | 0 | 26 |  | |
|  | Cork | 9 | 18 | 0 | 27 |  | |
|  |  | 18/0 | 35/4 | 0/0 | 53/4 | 7.6% | |
| United Kingdom | Glasgow | 19 | 18 | 0 | 38 |  | |
|  | Manchester | 21 | 19 | 0 | 40 |  | |
|  | Cardiff | 22 | 19 | 0 | 42 |  | |
|  | Plymouth | 20 | 16 | 0 | 36 |  | |
|  |  | 82/3 | 72/5 | 0/0 | 154/8 | 5.19% | |
| Germany | Hamburg | 35 | 23 | 0 | 58 |  | |
|  | Berlin | 20 | 0 | 0 | 20 |  | |
|  | Frankfurt | 9 | 0 | 0 | 9 |  | |
|  |  | 64/3 | 23/3 | 0/0 | 87/6 | 6.90% | |
|  |  | 225/15 | 268/21 | 52/1 | 545/37 | 6.79% | |

S1Table. Tuna samples collected in six EU countries between 2012 and 2014. Cities of sampling and type of tuna product are indicated for each country and city. Total number of samples in each category are indicated in black, mislabelled samples are reported after the slash, in red.
