# Supplementary material for: Tuna labels matter in Europe: Mislabelling rates in different tuna products
Source: PLoS One. 2018 May 16;13(5):e0196641. doi: 10.1371/journal.pone.0196641 (PMC5955508; doi:10.1371/journal.pone.0196641)
Supplement: S2 Table — (DOCX) [file pone.0196641.s002.docx]

S2 Table. Commercial denominations of tuna in EU and member states participating in this collaborative work.

.

| **Country** | **Legislation** | **commercial name** | **Species** |
| --- | --- | --- | --- |
|  | **Council regulation EU1379/2013** | Tuna | *Thunnus spp* |
|  |  |  | *Katsuwonus pelamis* |
|  |  | Bonito | *Sarda spp* |
|  |  |  | *Euthynnus spp* |
|  |  |  | *Auxis spp* |
| **Spain** | Council Regulation 1536/92 (canned tuna) | Atún/ Tuna | *Thunnus spp* |
|  |  |  | *Katsuwonus pelamis* |
|  |  |  | *Allothunnus fallai* |
|  | BOE Resolución 8/03/2017 Secretaria General de Pesca | Bonito | *Sarda sarda* |
|  |  | Bonito del Pacífico | *Sarda chiliensis* |
|  |  | Bonito oriental | *Sarda orientalis* |
|  | Real Decreto 1385/2009 | Atún o Atún claro | *Thunnus albacares* |
|  |  |  | *Thunnus obesus* |
|  | BOE Resolución 8/03/2017 Secretaria General de Pesca | Rabil, Atún de aleta amarilla | *Thunnus albacares* |
|  |  | Atún blanco, Bonito del Norte, Albacora | *Thunnus alalunga* |
|  |  | Atún de aleta negra | *Thunnus atlanticus* |
|  |  | Atún del Pacífico | *Thunnus orientalis* |
|  |  | Atún del Sur | *Thunnus maccoyii* |
|  |  | Atún lanzón | *Allothunnus fallai* |
|  |  | Atún rojo, Atún de aleta azul | *Thunnus thynnus* |
|  |  | Atún tongol, Tongol | *Thunnus tonggol* |
|  |  | Atún de ojo grande, Patudo, patudo del atlántico | *Thunnus obesus* |
|  |  | Listado, Bonito de vientre rayado | *Katsuwonus pelamis* |
|  |  | Bacoreta | *Euthynnus alletteratus* |
|  |  | Bacoreta oriental | *Euthynnus affinis* |
|  |  | Melva | *Auxis rochei* |
|  |  |  | *Auxis thazard* |
| **Portugal** | **Portaria n.º 587/2006, de 22 de Junho** | Atum | *Thunnus spp* |
|  |  |  | *Katsuwonus pelamis* |
|  |  | Bonito | *Scomberomorus spp* |
|  |  |  | *Acanthocybium solandri* |
|  |  |  | *Euthynnus alletteratus* |
|  |  |  | *Katsuwonus pelamis* |
|  |  |  | *Sarda spp.* |
|  |  | Atum-voador, Germão | *Thunnus alalunga* |
|  |  | Atum-albacora, Galha-a-ré, Galha-amarela | *Thunnus albacares* |
|  |  | Atum-patudo | *Thunnus obesus* |
|  |  | Atum-rabilho-do-Pacífico | *Thunnus orientalis* |
|  |  | Atum-rabilho, Rabilo, Rabil | *Thunnus thynnus* |
|  |  | Serra-da-Índia, Cavala-da-Índia | *Acanthocybium solandri* |
|  |  | Merma | *Euthynnus alletteratus* |
|  |  | Gaiado, Raiado | *Katsuwonus pelamis* |
|  |  | Sarrajão, Serrajão | *Sarda sarda* |
|  |  | Serra-espanhola | *Scomberomorus maculatus* |
|  |  | Judeu | *Auxis rochei* |
|  |  |  | *Auxis thazard* |
| **Germany** | **Verzeichnis der Handelsbezeichnungen für Erzeugnisse der Fischerei und Aquakultur 20.09.2013** | Thunfisch | *Thunnus spp* |
|  |  |  | *Katsuwonus pelamis* |
|  |  | Bonito | *Euthynnus spp* |
|  |  |  | *Gymnosarda spp.* |
|  |  |  | *Orcynopsis unicolor* |
|  |  |  | *Sarda spp* |
|  |  | Bonito, Pelamide | *Gymnosarda spp.* |
|  |  |  | *Sarda spp* |
|  |  | Bonito, Fregattmakrele | *Auxis spp.* |
|  |  | Echter Bonito | *Katsuwonus pelamis* |
|  |  | Weißer Thun, Germon | *Thunnus alalunga* |
|  |  | Gelbflossen-Thun | *Thunnus albacares* |
|  |  | Schwarzflossen-Thun | *Thunnus atlanticus* |
|  |  | Blauflossen-Thun | *Thunnus maccoyii* |
|  |  | Großaugen-Thun | *Thunnus obesus* |
|  |  | Roter Thun | *Thunnus thynnus* |
|  |  | Langschwanz-Thun | *Thunnus tonggol* |
| **France** | **Liste des dénominations commerciales -Poissons - 10/04/2013** | thon listao (cans) | *Katsuwonus pelamis* |
|  |  | germon, thon germon, thon blanc | *Thunnus alalunga* |
|  |  | albacore, thon albacore | *Thunnus albacares* |
|  |  | thon à nageoires noires | *Thunnus atlanticus* |
|  |  | thon rouge du sud | *Thunnus maccoyii* |
|  |  | thon obèse | *Thunnus obesus* |
|  |  | thon rouge | *Thunnus thynnus* |
|  |  | thonine orientale | *Euthynnus affinis* |
|  |  | thonine commune | *Euthynnus alletteratus* |
|  |  | bonite à ventre rayé, listao | *Katsuwonus pelamis* |
|  |  | *bonitou, melva* | *Auxis rochei rochei* |
|  |  | *auxide, melva* | Auxis thazard thazard |
| **UK** | **2013 No. 1768 Fish labelling regulations** | Tuna or Tunny | *Thunnus spp* |
|  |  |  | *Katsuwonus pelamis* |
|  |  | Bonito | *Sarda spp* |
|  |  |  | *Euthynnus spp* |
|  |  |  | *Auxis spp* |
|  |  | Bullet tuna or Melva | *Auxis rochei* |
|  |  | Skipjack tuna or Tuna | *Katsuwonus pelamis* |
|  |  | Albacore tuna | *Thunnus alalunga* |
|  |  | Bigeye tuna | *Thunnus obesus* |
|  |  | Bluefin tuna | *Thunnus thynnus* |
|  |  | Oriental bluefin tuna or Pacific bluefin tuna | *Thunnus orientalis* |
|  |  | Southern bluefin tuna | *Thunnus maccoyii* |
|  |  | Yellowfin tuna | *Thunnus albacares* |
| **Ireland** | **S.I. No. 320 of 2003** | Bullet tuna, Frigate and bullet tunas | *Auxis rochei rochei* |
|  |  | Frigate tuna, Frigate and bullet tunas | *Auxis thazard thazard* |
|  |  | Plain bonito | *Orcynopsis unicolor* |
|  |  | Striped bonito | *Sarda orientalis* |
|  |  | Atlantic bonito | *Sarda sarda* |
|  |  | Albacore | *Thunnus alalunga* |
|  |  | Yellowfin tuna | *Thunnus albacares* |
|  |  | Blackfin tuna | *Thunnus atlanticus* |
|  |  | Southern bluefin tuna | *Thunnus maccoyii* |
|  |  | Bigeye tuna | *Thunnus obesus* |
|  |  | Northern bluefin tuna | *Thunnus thynnus* |
|  |  | Longtail tuna | *Thunnus tonggol* |
