# Supplementary material for: Tuna labels matter in Europe: Mislabelling rates in different tuna products
Source: PLoS One. 2018 May 16;13(5):e0196641. doi: 10.1371/journal.pone.0196641 (PMC5955508; doi:10.1371/journal.pone.0196641)
Supplement: S3 Table — (DOCX) [file pone.0196641.s003.docx]

S3 Table. Methods and DNA target used by the participant laboratories in this study.

| **Country** | **DNA extraction** | **Target fragment** | **DNA sequencing** | **Sequence analysis** |
| --- | --- | --- | --- | --- |
| SPAIN | Wizard DNA Clean-up System Kit (Promega, WI USA) | - Cyt b-464bp (Burgener 1997) - Cyt b-176bp (Meyer 1993) - Cyt b-187bp (Mackie et al. 1999) | - Illustra Exostar 1-step (GE Healthcare, Buckinghamshire, UK) - Big dye Terminator 1.1 (Applied Biosystems, MA USA) - ABI Prism 310 (Aplied Biosystems) | - Chromas (Technelysium Pty Ltd.) - Bioedit (Hall 1999) - MEGA (Neighbor joining with IIM-CSIC reference sequences) (Kumar et al 2008) - BLAST (NCBI database) |
| UK-ROI | Chelex (Estoup et al. 1996) | - COI-650bp (Ward et al. 2005) - Cyt b-187bp (Mackie et al. 1999) | - Conducted by Beckman Coulter (Essex, UK) | - Bioedit (Hall 1999) - BOLD database (Ratnasingham and Hebert 2007) - BLAST (NCBI database) |
| PORTUGAL | DNeasy Blood and Tissue Kit (Qiagen, Hilden, Germany) | - Cyt b-464bp (Burgener 1997) - Cyt b-176bp (Meyer 1993) - Cyt b-187bp (Mackie et al. 1999) - COI-650bp | - Nzytec purification Kit (Nzytec, Lisbon, Portugal) - Sequencing reactions conducted by Biopremier (Lisbon, Portugal) | - Bioedit (Hall 1999) - BOLD database (Ratnasingham and Hebert 2007) - BLAST (NCBI database) |
| FRANCE | Charge Switch Forensic DNA purification Kit (Invitrogen, MA USA) | - CR-450bp (Viñas and Tudela 2009) - CR-450bp (Viñas and Tudela 2009) - CR-150bp (Mariani et al. 2015) | - Conducted by Genoscreen (Lille, France) | - Bioedit (Hall 1999) - MEGA (Neighbor joining with Ifremer reference sequences) (Kumar et al 2008) - BLAST (NCBI database) |
| GERMANY | CTAB (Rehbein et al., 2005) | - Cyt b-464bp (Burgener 1997) - Cyt b-176bp(Meyer 1993) - Cyt b-187bp (Mackie et al. 1999) | - Conducted by LGC Genomics (Berlin, Germany) | - Chromas (Technelysium Pty Ltd.) - GeneDoc (Nicholas, Nicholas & Deerfield 1997) - MEGA (Neighbor joining with IIM-CSIC reference sequences) (Kumar et al 2008) - BLAST (NCBI database) |
