# Supplementary material for: Tuna labels matter in Europe: Mislabelling rates in different tuna products
Source: PLoS One. 2018 May 16;13(5):e0196641. doi: 10.1371/journal.pone.0196641 (PMC5955508; doi:10.1371/journal.pone.0196641)
Supplement: S4 Table — (DOCX) [file pone.0196641.s004.docx]

S4 Table. Primers and reference for PCR protocol used in this study

| **DNA fragment** | **primer name** | **sequence** | **primer** | **PCR protocol** | **type of sample** | **used by** |
| --- | --- | --- | --- | --- | --- | --- |
| Cytb-464 bp | H15149ad | 5'-GCICCTCARAATGAYATTTGTCCTCA-3' | Burgener (1997) | Burgener (1997) | fresh, frozen | Spain, Portugal, Germany |
|  | L14735 | 5'AAAAACCACCGTTGTTATTCAACTA-3' |  |  |  |  |
| Cytb-176 bp | H15573 | 5'AATAGGAAGTATCATTCGGGTTTGATG-3' | Meyer (1993) | Meyer (1993) | processed | Spain, Portugal, Germany |
|  | L15424 | 5'ATCCCATTCCACCCATACTACTC-3' |  |  |  |  |
| Cytb-187 bp | BDR-L | 5’-GCMAACGGSGCNTCYTTCTTCTT-3’ | Mackie et al. (1999) | Mackie et al. (1999) | processed | Spain, Portugal, Germany, UK, ROI |
|  | BDR-H | 5’TGACGGTAGCHCCTCAGAADGACATTTGTCCTCA 3’ |  |  |  |  |
| COI-650 bp | FishF2 | 5′TCGACTAATCATAAAGATATCGGCAC3′ | Ward et al. (2005) | Griffiths et al. (2013) | fresh, frozen | UK, ROI, Portugal |
|  | FishR2 | 5′ACTTCAGGGTGACCGAAGAATCAGAA3′ |  |  |  |  |
| CR-400 bp | L15998 | 5′-TACCCCAAACTCCCAAAGCTA-3′ | Alvarado-Bremer (1994)  Mariani et al., 2015 | Viñas and Tudela (2009) | fresh, frozen | France |
|  | DLtun360 | 5'-TGTCCCTCACCTTCAATAACCG -3' |  |  |  |  |
| CR-150 bp | DLtun238 | 5'-CGAGATTTAAGACCTACCA - 3' | Mariani et al., 2015 | Viñas and Tudela (2009) | processed |  |
|  | DLtun360 | 5'-TGTCCCTCACCTTCAATAACCG -3' | Mariani et al., 2015 | Viñas and Tudela (2009) | Processed (canned) |  |
