# Supplementary material for: Tuna labels matter in Europe: Mislabelling rates in different tuna products
Source: PLoS One. 2018 May 16;13(5):e0196641. doi: 10.1371/journal.pone.0196641 (PMC5955508; doi:10.1371/journal.pone.0196641)
Supplement: S5 Table — (DOCX) [file pone.0196641.s005.docx]

S5 Table. Number of samples of fresh and frozen tuna analysed and mislabelling results split by type of label.

| Country | Label | Nº Samples (%) | Mislabelled (%) |
| --- | --- | --- | --- |
| Spain | Tuna | 9 (45%) | 0 (0%) |
|  | Species indicated | 11 (55%) | 5 (45%) |
|  | Bluefin (*T.thynnus*, BFT) | 4 (20%) | 4 (100%) |
| Portugal | Tuna | 4 (29%) | 0 (0%) |
|  | Species indicated | 10 (71%) | 3 (30%) |
|  | BFT | 1 (7%) | 1 (100%) |
| France | Tuna | 9 (35%) | 0 (0%) |
|  | Species indicated | 17(65%) | 4 (23.5%) |
|  | BFT | 2 (4%) | 1 (50%) |
| ROI | Tuna | 18 (100%) | 0(0%) |
|  | Species indicated | 0 (0%) | N.A. |
|  | BFT | 0 (0%) | N.A. |
| UK | Tuna | 31 (37%) | 0 (0%) |
|  | Species indicated | 53 (63%) | 5 (9.4%) |
|  | BFT | 0 (0%) | N.A. |
| Germany | Tuna | 26 (39%) | 1 (3,9%) |
|  | Species indicated | 40 (61%) | 7 (17.5%) |
|  | BFT | 1 (1.5%) | 1 (100%) |
| GLOBAL | Tuna | 97 (43%) | 1 (0.4%) |
|  | Species indicated | 131 (61%) | 7 (17.5%) |
|  | BFT | 8 (3.5%) | 7 (87.5%) |

Nº samples (%): the percentage is calculated with the total number of fresh and frozen samples in each country

Mislabelling (%): the percentage is referenced to the number of samples in each category label (i.e. 5 mislabelled samples in 11= 45%).

N.A. no samples taken with ABFT label.
