# Supplementary material for: Tuna labels matter in Europe: Mislabelling rates in different tuna products
Source: PLoS One. 2018 May 16;13(5):e0196641. doi: 10.1371/journal.pone.0196641 (PMC5955508; doi:10.1371/journal.pone.0196641)
Supplement: S6 Table — (DOCX) [file pone.0196641.s006.docx]

S6 Table. Number of samples of canned tuna analysed and mislabelling results split by type of label.

| Country | Label | Nº Samples (%) | Mislabelled(%) |
| --- | --- | --- | --- |
| Spain N=56 | Tuna | 9 (16%) | 0 (0%) |
|  | Species (*T. albacares*/ *T.obesus*) | 47 (84%) | 6 (13%) |
| Portugal N=53 | Tuna | 47 (89%) | 1 (2%) |
|  | Species (Yellowfin/Skipjack) | 6 (11%) | 1 (17%) |
| France N=29 | Tuna | 11 (38%) | 0 (0%) |
|  | Species (*T. albacares*) | 6 (21%) | 1 (17%) |
|  | Species (*T. alalunga*) | 8 (28%) | 0 (0%) |
|  | Species (*K.pelamis*) | 3(10%) | 0 (0%) |
|  | Species (*T. obesus*) | 1 (3%) | 0 (0%) |
|  | Species | 18 (62%) | 1 (6%) |
| ROI N=35 | Tuna | 3 (9%) | 0(0%) |
|  | Species (*K. pelamis*) | 23 (66%) | 2(9%) |
|  | Species (*T. albacares*) | 9 (26%) | 2(22%) |
|  | Species | 32 (91%) | 4(13%) |
| UK N=72 | Tuna | 0 (0%) | N.A. |
|  | Species (*K. pelamis*) | 48 (66%) | 5 (10%) |
|  | Species (*T. albacares*) | 18 (25%) | 0 (0%) |
|  | Species (*T. alalunga*) | 6 (25%) | 0 (0%) |
|  | Species | 72 (100%) | 5 (7%) |
| Germany N=23 | Tuna | 1 (4%) | 0(0%) |
|  | Species (*T. albacares*) | 4 (17%) | 0 (0%) |
|  | Species (*T. alalunga*) | 5 (22%) | 3 (60%) |
|  | Species (*K. pelamis*) | 13 (57%) | 0 (0%) |
|  | Species | 22 (96%) | 3 (13%) |
| GLOBAL N=268 | Tuna | 70 (26%) | 1 (1%) |
|  | Species indicated | 198 (74%) | 20 (10%) |

Nº of samples (%): the percentage is calculated with the total number of canned samples in each country

Mislabelling (%): the percentage is referenced to the number of samples in each category label (i.e. 6 mislabelled samples in 47= 13%).

N.A. This laboratory did not analysed samples labelled as “Tuna”, only samples with specific names.
