# Supplementary material for: Tuna labels matter in Europe: Mislabelling rates in different tuna products
Source: PLoS One. 2018 May 16;13(5):e0196641. doi: 10.1371/journal.pone.0196641 (PMC5955508; doi:10.1371/journal.pone.0196641)
Supplement: S7 Table — (DOCX) [file pone.0196641.s007.docx]

S7 Table. Species which have appeared in the mislabelled samples of this study

**Fresh and Frozen Samples**

| labeled as | identified as | number of samples |
| --- | --- | --- |
| Thunnus albacares/t Yellowfin / Thon albacore/  N=101, Mislabelled=12, Mislab.%=12% | *Thunnus obesus* | 12/12 (100%) |
| Thunnus thynnus/Bluefin Tuna/ Thon Rouge/ Atún Rojo N=8, Mislabelled=7, Mislab.%=88% | *Thunnus albacares* | 3/7 (43%) |
|  | *Thunnus obesus* | 3/7 (43%) |
|  | *T. atlanticus* | 1/7 (14%) |

**Canned Samples**

| labeled as | identified as | number of samples |
| --- | --- | --- |
| Thunnus albacares/t Yellowfin / Thon albacore/Atún Claro  N=86, Mislabelled=10, Mislab.%=12% | *Thunnus alalunga* | 5/10 (50%) |
|  | *Thunnus obesus* | 2/10 (20%) |
|  | *Katsuwonus pelamis* | 2/10 (20%) |
|  | *Euthynnus alleteratus* | 1/10 (10%) |
| Katsuwonus pelamis/Skipjack (UK samples) N=72, Mislabelled=7, Mislab.%=10% | *Thunnus albacares/T. obesus* | 7/7 (100%) |
| Thunnus alalunga (Germany Samples)N=19, Mislabelled=3, Mislab.%=16% | *Katsuwonus pelamis* | 3/3 (100%) |
