# Supplementary material for: Tuna labels matter in Europe: Mislabelling rates in different tuna products
Source: PLoS One. 2018 May 16;13(5):e0196641. doi: 10.1371/journal.pone.0196641 (PMC5955508; doi:10.1371/journal.pone.0196641)
Supplement: S1 File — (DOCX) [file pone.0196641.s008.docx]

**Tuna Species Identification Ring Trials**

The laboratories participating in the present study performed a series of ring trials to show that the methods employed by each of them were producing the same results.

Reference tissues belonging to the eight *Thunnus* species were analysed with different DNA markers and sequencing which afterwards were employed for the analysis of the commercial samples. Essentially two approaches were used, homology analysis and distance genetic measurement with phylogenetic tree construction (Forensically Informative Nucleotide Sequencing, FINS). The methods tested were COI sequencing (barcoding with homology search) (UK), Cytochrome b FINS (Spain and Germany) and Control Region FINS (France) with the primers and protocols described in S3 Table. The species were identified as those with the highest match in the corresponding reference database, or those forming a monophyletic group upon phylogenetic analysis. In situations where different species presented the same percent homology match or the phylogenetic tree was ambiguous this result was recorded.

All tuna samples were unequivocally differentiated whether the homology approach or the phylogenetic tree were used (see table below), with the exception of *Thunnus albacares* which resulted undistinguishable from *Thunnus obesus* with the COI method. The problems to correctly identify the species level of *Thunnus albacares*, were expected beforehand, since phylogenetic studies have shown that due to recent divergence of *Thunnus albacares* and *Thunnus obesus* differentiation with mitochondrial markers is not possible using COI marker (Hanner et al., 2011, Pedrosa-Gerasmio et al. 2012). Hence, the COI gen is unable to fully resolve these two species (Lowenstein et al. 2009). Consequently, it was decided not to consider the substitution between these two species as mislabelling when the homology percent obtained was lower than 100%, and the same criterion was applied to the samples analysed with the rest of methods.

In the table below we can observe some differences between homology and genetic distance method results., whereas with genetic distance methods identification was always successful and coherent with the reference sample, homology results were not always reliable. The ability of a database to identify a sample depends on the size and accuracy of the database, among other reasons. In the case of Genbank, which is by far the largest DNA sequence data base in the world, it has to be considered that it has not been designed for species identification and therefore some inconsistencies may be found regarding terminology for particular species, besides it does not guarantee the absence of errors due to lack of voucher specimens (Hellberg et al., 2016).

This ring trial revealed the higher reliability of FINS, which solves the identification problems of homology-based methods by using genetic distances and phylogenetic tree. Therefore, in light of these results, all three tested methods appeared to be applicable for the objectives of the study and led authors to be confident in cross-lab consistency and reproducibility of the data obtained.

**Comparison of methodologies for species identification.** For each sample the percentage of species match is presented. In case the method could not unambiguously distinguish between two or more species, the other alternative match is also given.

| **Species** | **COI sequencing (UK)** | | **Cyt b FINS (Spain)** | | **Control Region FINS (France)** | | **Cyt b FINS (Germany)** | |
| --- | --- | --- | --- | --- | --- | --- | --- | --- |
|  | **BOLD Tree ID** | **BOLD Match** | **NJ Tree ID** | **NCBI Match** | **NJ Tree ID** | **NCBI Match** | **NJ Tree ID** | **NCBI Match** |
| ***Thunnus maccoyii*** | *Thunnus maccoyii* | *T. maccoyii 99.84%* | *Thunnus maccoyii* | *Thunnus maccoyii 100%* | No reference | *T. maccoyii 87%* | *Thunnus maccoyii* | *T. maccoyii 100 %* |
| ***Thunnus orientalis*** | *Thunnus orientalis* | *T. orientalis 100%* | *Thunnus orientalis* | *Thunnus orientalis/T.alalunga 99%* | No reference | *T. orientalis 97%* | *Thunnus orientalis* | *T. orientalus/T. thynnus 100 %)* |
| ***Thunnus alalunga*** | *Thunnus alalunga* | *T.alalunga 100%* | *Thunnus alalunga* | *T. alalunga 100%* | *Thunnus alalunga* | *T. alalunga 97%* | *Thunnus alalunga* | *T. alalunga/T. thynnus 100* |
| ***Thunnus albacares*** | *Thunnus albacares/T. obesus* | *T.albacares/T. obesus 100%* | *Thunnus albacares* | *T. albacares 100%* | *Thunnus albacares* | *T. albacares 99%* | *Thunnus albacares* | *T. albacares/obesus 100 %* |
| ***Thunnus obesus*** | *Thunnus obesus* | *T. obesus 100%* | *Thunnus obesus* | *T. obesus100%* | *Thunnus obesus* | *T. obesus 99%* | *Thunnus obesus* | *T. obesus 100 %* |
| ***Thunnus thynnus*** | *Thunnus thynnus* | *T. thynnus 100%* | *Thunnus thynnus* | *T. thynnus 100%* | *Thunnus thynnus* | *T. thynnus 99%* | *Thunnus thynnus* | *T. thynnus 100 %* |
| ***Thunnus tonggol*** | *Thunnus tonggol* | *T. tonggol 100%* | *Thunnus tonggol* | *T. tonggol/T. atlanticus/T.albacares 99%* | No reference | *T. tonggol 98%* | *Thunnus tonggol* | *T. tonggol 99 %* |
| ***Katsuwonus pelamis*** | *Katsuwonus pelamis* | *K. pelamis 100%* | *Katsuwonus pelamis* | *Katsuwonus pelamis 99%* | *Katsuwonus pelamis* | *K. pelamis 99%* | *Katsuwonus pelamis* | *K. pelamis 100 %* |

References:

- Hanner R, Becker S, Ivanova N V., Steinke D. FISH-BOL and seafood identification: Geographically dispersed case studies reveal systemic market substitution across Canada. Mitochondrial DNA. 2011;22: 106–122.
- Pedrosa-Gerasmio IR, Babaran RP, Santos MD (2012) Correction: Discrimination of Juvenile Yellowfin (Thunnus albacares) and Bigeye (T. obesus) Tunas using Mitochondrial DNA Control Region and Liver Morphology. Plos One e35604
- Lowenstein JH, Amato G, Kolokotronis S. The real maccoyii: identifying tuna sushi with DNA barcodes – contrasting characteristic attributes and genetic distances (2009). PlosOne e7866.
- Hellberg RS, Pollack S, Hanner RH. 2016. Seafood species identification using DNA sequencing. In “Seafood authenticity and traceability: A DNA perspective”. Naaum A.M., Hanner R.H. Academic Press
